# Association between the atherogenic index of plasma and major adverse cardiovascular events among non-diabetic hypertensive older adults

**DOI:** 10.1186/s12944-022-01670-6

**Published:** 2022-07-22

**Authors:** Fei Hang, Jieruo Chen, Zefeng Wang, Keyang Zheng, Yongquan Wu

**Affiliations:** Department of Cardiology, Beijing Anzhen hospital, Capital Medical University, No.2 Anzhen Road, Beijing, 100029 China

**Keywords:** Atherogenic index of plasma, Major adverse cardiovascular events, Hypertension, Intensive blood pressure management

## Abstract

**Background:**

Literature on the association between the atherogenic index of plasma (AIP) and the risk of major adverse cardiovascular events (MACEs) among non-diabetic hypertensive older adults is quite limited.

**Methods:**

A post-hoc analysis of data obtained from the Systolic Blood Pressure Intervention Trial was performed. The predictive value of AIP on the risk of MACEs among non-diabetic hypertensive older adults was assessed to evaluate whether the benefit of intensive blood pressure (BP) control in preventing MACEs is consistent in different AIP subgroups.

**Results:**

In this study, 9323 participants with AIP were included, out of which 561 (6.02%) had composite cardiovascular outcomes during a median of 3.22 years of follow-up. Patients in the highest AIP quartile had a significantly increased risk of the primary outcome. In the fully adjusted Model 3, the adjusted hazard ratios (HRs) of the primary outcome for participants in Q2, Q3, and Q4 of AIP were 1.32 (1.02, 1.72), 1.38 (1.05, 1.81), and 1.56 (1.17, 2.08) respectively. Consistently, the trend test for the association between AIP quartiles and the primary outcome showed that a higher AIP quartile was associated with a significantly higher risk of the primary outcome (adjusted HR (95%CI) in model 3:1.14 (1.04, 1.25), *P* = 0,004). However, within each AIP quartile, absolute event rates were lower in the intensive treatment group. No evidence was found for the interaction between intensive BP control and AIP for the risk of the primary outcome (*P* for interaction = 0.932).

**Conclusion:**

This study found that elevated AIP was independently and positively associated with the risk of MACEs among non-diabetic hypertensive older adults. The benefits of intensive BP control in managing cardiovascular events were consistent in different AIP subgroups.

**Supplementary Information:**

The online version contains supplementary material available at 10.1186/s12944-022-01670-6.

## Background

Dyslipidemia is one of the known risk factors for cardiovascular diseases, which are the main cause of morbidity and mortality worldwide [1]. A number of blood lipid parameters have been used to predict the risk of cardiovascular outcomes, including total cholesterol, triglyceride (TG), low-density lipoprotein cholesterol (LDL-C), and high-density lipoprotein cholesterol (HDL-C) [2–5].

Small, dense low-density lipoprotein (sdLDL) subfractions were found to be proatherogenic and were highly related to carotid artery stenosis [6, 7]. Therefore, in order to estimate the risk of atherosclerosis accurately, it is recommended to measure lipoprotein particle size distribution with gradient gel electrophoresis (GGE); however, this is not routinely performed in patients with hypertension [8]. The atherogenic index of plasma (AIP), calculated using the logarithm of the ratio between the level of TG and HDL-C, is an indicator reflecting the characteristics and the degree of abnormal lipid metabolism [9]; additionally, it has been widely used as a surrogate for sdLDL in assessing plasma atherogenicity [9]. Previous studies revealed that AIP was associated with the risks of several cardiovascular diseases, such as incident ischemic heart disease, atherosclerosis, coronary artery disease, acute ischemic stroke, etc. [10–12]. However, previous studies have mainly focused on the general population. To our knowledge, information on the association between AIP and the risk of major adverse cardiovascular events (MACEs) among non-diabetic hypertensive older adults (age ≥ 50 years) is very limited.

Upon comparison of patients’ results from the Systolic Blood Pressure Intervention Trial (SPRINT) with those of the standard blood pressure (BP) control (a systolic BP control target of less than 140 mmHg), intensive BP management (a systolic BP control target of less than 120 mmHg) revealed significantly better cardiovascular outcomes among non-diabetic hypertensive patients with an increased risk of cardiovascular events.^13^ Based on the intensive blood pressure treatment proposed by SPRINT, many patients may adopt this treatment in the future clinical work. However, it is less known whether the association between AIP and MACEs is independent of standard or intensive BP control. Therefore, this study aimed to explore the predictive value of AIP on the risk of MACEs among non-diabetic hypertensive older adults and evaluate whether the benefit of intensive BP control in preventing MACEs is consistent in different AIP subgroups.

## Methods

### Study population and design

The data used in this study were derived from the SPRINT dataset, available at the National Heart, Lung, and Blood Institute data repository in the Biologic Specimen and Data Repository Information Coordinating Center. SPRINT was a randomized, controlled, open-label trial conducted at 102 clinical sites in the United States and was approved by the institutional review boards of the participating centers. The protocol and main outcomes have previously been published [13, 14]. The SPRINT study included patients who were at least 50 years old, had systolic BP levels of 130–180 mmHg, and had an increased risk of cardiovascular disease. Patients with diabetes, polycystic kidney disease, or a previous stroke were excluded. The primary outcome of this study was that in patients with hypertension and high cardiovascular risk, intensive BP control could significantly reduce the rate of composite cardiovascular outcomes and all-cause mortality compared with standard BP control.

The main aim of this analysis was to assess the association between the baseline AIP (calculated by TG and HDL-C) and MACEs in SPRINT participants, and to investigate whether AIP modifies the benefit of intensive BP control in preventing MACEs. 38 participants were excluded with missing baseline TG and HDL-C levels, and 9323 participants were included in the final analysis.

### Evaluation of atherogenic index of plasma and study outcomes

The atherogenic index of plasma was calculated using the base 10 logarithm of the TG/HDL-C mole ratio. The adopted conversion factors were TG1 ng/dL = 0.011 mmol/L and HDL-C1 ng/dL = 0.026 mmol/L. The selected participants (9323) were grouped according to AIP quartiles: Q1 (AIP ≤ -0.228, *n* = 2331), Q2 (− 0.228 < AIP ≤ -0.037, *n* = 2329), Q3 (− 0.037 < AIP ≤ 0.156, *n* = 2330), and Q4 (AIP > 0.156, *n* = 2333). The Q1 group was used as reference.

The primary outcomes of this analysis were composite cardiovascular outcomes, including myocardial infarction (MI), non-MI acute coronary syndrome, stroke, cardiovascular mortality, and heart failure. The definition of outcomes was published in the SPRINT protocol.^14^

### Statistical analysis

Participants were grouped according to the AIP quartiles. Continuous variables were expressed as mean (standard deviation) or median (Q1-Q3) based on the distribution of data. The differences between the quartiles were tested using ANOVA and the Kruskal-Wallis H test for normal distribution data and skewed distribution data, respectively. All categorical variables were expressed as frequencies (percentiles). The chi-square test or Fisher’s exact test was used to test the differences in categorical variables between groups.

Cox model was used to assess the association between AIP quartiles and occurrence of the primary outcome in the three models. Model 1 was adjusted to none. Model 2 was adjusted for age, race, and the treatment arm. Model 3 was adjusted for age, race, treatment arm, body mass index, systolic BP, heart rate, smoking status, serum creatinine, fasting total cholesterol, fasting glucose, previous cardiovascular disease (CVD), previous chronic kidney disease (CKD), aspirin use, and statin use. The Schoenfeld residual test was used to test the proportional hazard assumption in the Cox model. The robustness of the association between the AIP and the primary outcome was evaluated in the prescribed subgroups using subgroup analysis and interaction tests. To establish the relationship between AIP and the effect of intensive BP control on the risk of the primary outcome, *P* value for interaction was calculated. Model 3 was adjusted for all covariates, except for the treatment arm. Poisson regression was used to assess the incidence of primary outcomes by treatment arm and AIP quartile.

All analyses were performed using the statistical software package R (The R Foundation; http://www.R-project.org). Statistical significance was set at *P* < 0.05.

## Results

### Baseline characteristics of the participants

A total of 9323 participants with AIP were included in this analysis, and 561 (6.02%) had composite cardiovascular outcomes during a median of 3.22 years of follow-up. The baseline characteristic of the included participants based on the AIP quartiles are shown in Table 1. Participants in the higher quartiles of AIP had a higher body mass index, diastolic BP, heart rate, serum creatinine, fasting TGs, glucose, and cardiovascular risk than those in the lower quartiles. Participants in the higher quartiles were more likely to be < 75 years of age, male, and current smokers. Compared with participants in lower quartiles, those in the higher quartiles were more likely to have CKD and a higher rate of composite cardiovascular outcomes.

**Table 1:** Baseline characteristic of the participants included in the analysis according to AIP quartiles

Variable	AIP				*P* value
	Q1	Q2	Q3	Q4	
N	2331	2329	2330	2333	
AIP, mean (SD)	−0.39 (0.12)	− 0.13 (0.05)	0.05 (0.06)	0.35 (0.17)	< 0.001
Female, n (%)	1056 (45.30%)	891 (38.26%)	760 (32.62%)	600 (25.72%)	< 0.001
Treatment					
Intensive, n (%)	1168 (50.11%)	1173 (50.36%)	1171 (50.26%)	1150 (49.29%)	0.882
BMI (Kg/m2), median (Q1-Q3)	27.02 (24.05–30.87)	28.59 (25.69–32.40)	29.63 (26.69–33.49)	30.42 (27.52–34.14)	< 0.001
Age, y					
Overall	70.01 (9.53)	68.44 (9.41)	67.66 (9.19)	65.51 (8.98)	< 0.001
≥ 75y, n (%)	871 (37.37%)	700 (30.06%)	615 (26.39%)	455 (19.50%)	< 0.001
Race, n (%)					< 0.001
Non-Hispanic White	1207 (51.78%)	1303 (55.95%)	1382 (59.31%)	1494 (64.04%)	
Non-Hispanic Black	919 (39.43%)	790 (33.92%)	622 (26.70%)	454 (19.46%)	
Hispanic	160 (6.86%)	201 (8.63%)	280 (12.02%)	337 (14.44%)	
Other	45 (1.93%)	35 (1.50%)	46 (1.97%)	48 (2.06%)	
Baseline BP, mm Hg					
Systolic (mm Hg)	140.94 (16.03)	139.82 (15.52)	139.20 (15.58)	138.71 (15.09)	< 0.001
Diastolic (mm Hg)	77.15 (12.42)	78.00 (11.83)	77.85 (11.45)	79.51 (11.93)	< 0.001
Distribution of systolic BP, n (%)					< 0.001
≤ 132 mmHg	712 (30.54%)	792 (34.01%)	798 (34.25%)	822 (35.23%)	
> 132 to < 145 mmHg	738 (31.66%)	738 (31.69%)	771 (33.09%)	779 (33.39%)	
≥ 145 mmHg	881 (37.79%)	799 (34.31%)	761 (32.66%)	732 (31.38%)	
Baseline heart rate, bpm	65.39 (11.33)	65.79 (11.44)	66.11 (11.72)	67.61 (11.82)	< 0.001
Serum creatinine, mg/dL	1.04 (0.32)	1.07 (0.32)	1.07 (0.34)	1.12 (0.37)	< 0.001
Urine Albumin/Creatinine ratio, mg/g Cr, median (Q1-Q3)	9.84 (5.86–20.75)	9.09 (5.49–19.97)	9.33 (5.62–22.82)	9.71 (5.57–23.00)	0.047
Estimated GFR, mL.min − 1 1.73 m − 2, median (Q1-Q3)	72.32 (59.75–86.22)	70.92 (57.90–83.91)	71.84 (58.38–84.95)	70.17 (56.49–83.44)	< 0.001
Fasting total cholesterol, mg/dL, median (Q1-Q3)	187.00 (162.00–214.00)	183.00 (158.00–209.00)	185.00 (158.00–212.00)	193.00 (168.00–223.00)	< 0.001
Fasting total TGs, mg/dL, median (Q1-Q3)	63.00 (54.00–74.00)	90.00 (80.00–104.00)	123.00 (109.00–138.00)	194.00 (162.00–242.00)	< 0.001
Fasting HDL-C, mg/dL, median (Q1-Q3)	66.00 (57.00–76.00)	53.00 (48.00–60.00)	47.00 (42.00–53.00)	40.00 (36.00–46.00)	< 0.001
Fasting glucose, mg/dL, median (Q1-Q3)	95.00 (89.00–101.00)	96.00 (90.00–104.00)	98.00 (91.00–106.00)	100.00 (93.00–109.00)	< 0.001
Statin use, n (%)	916 (39.65%)	1042 (44.99%)	1081 (46.76%)	1007 (43.33%)	< 0.001
Aspirin use, n (%)	1182 (50.86%)	1197 (51.42%)	1224 (52.65%)	1144 (49.16%)	0.120
TG-lowering drug, n (%)	533 (22.88%)	576 (24.73%)	653 (28.03%)	619 (26.53%)	< 0.001
Smoking status, n (%)					0.006
Never smoked	1088 (46.68%)	1031 (44.27%)	1024 (43.95%)	968 (41.49%)	
Former smoker	964 (41.36%)	994 (42.68%)	1006 (43.18%)	999 (42.82%)	
Current smoker	276 (11.84%)	302 (12.97%)	296 (12.70%)	364 (15.60%)	
Framingham 10-y CVD risk score, %, median (Q1-Q3)	14.71 (10.27–21.61)	16.80 (11.33–24.24)	18.49 (12.68–26.77)	21.39 (15.08–30.26)	< 0.001
Previous CVD, n (%)	420 (18.02%)	466 (20.01%)	502 (21.55%)	481 (20.62%)	0.021
Previous CKD, n (%)	596 (25.57%)	673 (28.90%)	641 (27.51%)	735 (31.50%)	< 0.001
Primary outcome	103 (4.42%)	137 (5.88%)	150 (6.44%)	171 (7.33%)	< 0.001

### Relationship between atherogenic index of plasma and primary outcome

Table 2 presents the hazard ratios (HRs) (95%CI) of the primary outcome among the included participants grouped by quartiles of AIP. Participants in the highest AIP quartile had a significantly increased risk of the primary outcome. This association persisted even after adjusting for potential confounding factors, including age, race, treatment arm, body mass index, systolic blood pressure, heart rate, smoking status, serum creatinine, fasting total cholesterol, fasting glucose, previous CVD, previous CKD, aspirin use, and statin use. In the fully adjusted model 3, the adjusted HRs of the primary outcome for participants in Q2, Q3, and Q4 of AIP were 1.32 (1.02, 1.72), 1.38 (1.05, 1.81), and 1.56 (1.17, 2.08) respectively. Consistently, the trend test for the association between AIP quartiles and the primary outcome showed that a higher AIP quartile was associated with a significantly higher risk of the primary outcome (adjusted HR (95%CI) in model 3: 1.14 [1.04, 1.25], *P* = 0.004).

**Table 2:** Association between AIP and primary outcome in different models

AIP quartiles	Model 1	Model 2	Model 3
	HR (95%CI) *P* value		
1	Ref.	Ref.	Ref.
2	1.33 (1.03, 1.72) *P* = 0.027	1.46 (1.13, 1.89) *P* = 0.004	1.32 (1.02, 1.72) *P* = 0.037
3	1.45 (1.13, 1.86) *P* = 0.004	1.68 (1.31, 2.17) *P* < 0.001	1.38 (1.05, 1.81) *P* = 0.020
4	1.65 (1.29, 2.10) *P* < 0.001	2.16 (1.68, 2.78) *P* < 0.001	1.56 (1.17, 2.08) *P* = 0.003
*P* for trend (1 Q increment)	1.16 (1.08, 1.25) *P* < 0.001	1.27 (1.18, 1.38) *P* < 0.001	1.14 (1.04, 1.25) *P* = 0.004

Model 1 were adjusted for none. Model 2 were adjusted for age, race and treatment arm. Model 3 were adjusted for age, race, treatment arm, body mass index, systolic blood pressure, heart rate, smoking status, serum creatinine, fasting total cholesterol, fasting glucose, previous CVD, previous CKD, aspirin use and statin use

### Subgroup analysis for the risk of primary outcome by baseline atherogenic index of plasma quartiles

Stratified analyses were performed to assess the impact of AIP (per 1 quartile increment) on the risk of the primary outcome. As shown in Table 3, the relationship between AIP and the primary outcome was consistent in the prescribed stratifications: sex (male vs. female), age (< 75 years vs. ≥75 years), ethnicity (black vs. non-black), previous CVD (yes vs. no), previous CKD (yes vs. no), Framingham 10-y CVD risk (< 15% vs. ≥15%), aspirin use (yes vs. no), and statin use (yes vs. no). All *P* for interaction was > 0.05.

**Table 3:** Subgroup analysis for the risk of primary outcome by baseline AIP quartiles (1 quartile increment)

	AIP Quartiles (1 Q increment)		
Subgroup	HR, 95%CI	*P* value	*P* for interaction
Sex			0.336
Male	1.67 (1.11, 2.52)	0.014	
Female	1.28 (0.80, 2.05)	0.305	
Age group			0.816
< 75	1.31 (0.89, 1.92)	0.174	
≥ 75	1.39 (0.91, 2.11)	0.129	
Race			0.398
Black	1.34 (0.77, 2.31)	0.302	
No-black	1.72 (1.20, 2.47)		
Previous CVD			0.932
Yes	1.62 (1.01, 2.58)	0.044	
No	1.65 (1.14, 2.40)	0.008	
Previous CKD			0.981
Yes	1.65 (1.04, 2.61)	0.034	
No	1.64 (1.13, 2.38)	0.01	
CVD risk			0.423
< 15%	1.47 (0.88, 2.46)	0.141	
≥ 15%	1.86 (1.27, 2.72)	0.001	
Aspirin use			0.912
Yes	1.66 (1.13, 2.43)	0.009	
No	1.61 (1.03, 2.52)	0.035	
Statin use			0.350
Yes	1.84 (1.22, 2.77)	0.003	
No	1.45 (0.96, 2.19)	0.074	

Model was adjusted for all covariates in Model 3 except stratifications itself

### Intensive blood pressure control and primary outcome according to atherogenic index of plasma quartiles

**Fig. 1:**
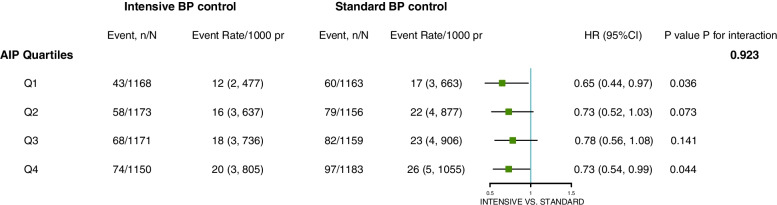
Intensive BP control and primary outcome according to AIP quartiles. *pr: person year. Model was adjusted for all covariates in Model 3 except treatment arm

The results stratified by AIP quartiles showed higher event rates with increasing frailty in both treatment groups (Fig. 1). However, within each AIP quartile, absolute event rates were lower in the intensive treatment group, compared to that of the standard control group. No evidence was found for the interaction between intensive BP control and AIP for the risk of the primary outcome (*P* for interaction = 0.932).

## Discussion

This secondary analysis of the SPRINT data revealed the association of higher AIP with a significantly higher risk of composite cardiovascular outcomes in hypertensive older adults. The benefits of intensive BP control on composite cardiovascular outcomes were consistent across different AIP quartiles. No evidence was found for the interaction between intensive BP control and AIP on the risk of the primary outcome.

A previous study found that the predominance of sdLDL in plasma was significantly associated with an increased risk of coronary heart disease [15]. Compared with larger and looser LDL particles, sdLDL particles are more likely to penetrate the artery wall and deposit in the vascular endothelium [16]. In addition, sdLDL are more susceptible to oxidation, which further increases atherogenicity [17]. Therefore, the relative proportion of sdLDL may be an important predictive factor of cardiovascular outcomes. However, LDL subclass patterns are usually determined by non-denaturing polyacrylamide GGE, which is ideally not performed when patients are admitted to a hospital [8]. Dobiasova et al. proposed AIP as an indicator of atherogenic lipoprotein phenotype and demonstrated its negative correlation with LDL particle size, making AIP a surrogate for sdLDL in predicting the atherogenicity of plasma lipoproteins [9].

Previous studies have reported a positive association between AIP and the risk of MACEs in different populations. A 15-year cohort study on healthy adults revealed the value of AIP in the prediction of developing cardiovascular events and its related mortality [18]. The results of a study on the Iranian population indicated that AIP was positively associated with the risk of cardiovascular diseases [19]. A cohort study on postmenopausal Chinese women (aged over 50 years) concluded that AIP was an independent predictor of cardiovascular risk. A study on the Health Risk Assessment Study and Korea Health Insurance Review and Assessment Service cohort found a positive correlation between the AIP quartile and the incidence of ischemic heart disease [10]. Older adults with a higher baseline BP had a higher Framingham score (a sex-specific algorithm for predicting the 10-year cardiovascular disease risk of an individual [20]), which represented a higher cardiovascular risk. Although a large body of evidence has proven the positive association between AIP and the risk of MACEs, this association has not been fully elucidated and needs to be studied in older adults with hypertension. Consistent with previous studies, this study found that elevated AIP was positively associated with a higher risk of MACEs in non-diabetic hypertensive older adults, and that AIP (Area Under Curve: 0.703 vs. 0.669, *P* < 0.001) was an efficient biomarker than LDL in predicting the incidence of MACEs (Fig. S1).

TG level was suggested to be positively associated with a higher prevalence of cardiovascular outcomes [21]. Within the high TG level group, patients with low HDL-C levels showed a higher risk of cardiovascular disease than those with high HDL-C levels [22]. Both increased TG levels and decreased HDL-C levels are strong indicators of cardiovascular risk. This mechanism may explain why elevated AIP, as a parameter of blood lipid levels, is strongly associated with an increased risk of MACEs. In addition, a previous study found that AIP levels were correlated with abnormal glucose metabolism, inferred from the degree of insulin resistance [23]. Insulin resistance, and related hyperinsulinemia, hyperglycemia, and adipocytokines may also cause vascular endothelial dysfunction, dyslipidemia, hypertension, and vascular inflammation, all of which contribute to the development of cardiovascular diseases.

According to a 9-year longitudinal study in Taiwan, AIP was proven to be significantly and positively correlated with the prevalence of hypertension [24]. A longitudinal study following a cohort of Hanzhong hypertensive adolescents for 12 years found that a high AIP level was a strong risk factor for hypertension-associated renal damage [25]. With a growing body of evidence showing a linear relationship between BP and the risk of cardiovascular diseases, the benefits of intensive BP control in decreasing MACEs risks have already been proven in older patients with hypertension [26, 27]. Therefore, this study evaluated the relationship between AIP and the effect of intensive BP management on the risk of cardiovascular diseases and found that the benefit of intensive BP control is consistent in different AIP quartiles. Additionally, no interaction between intensive BP control and AIP for the risk of primary outcome was found.

### Comparisons with other studies and what does the current work add to the existing knowledge

Previous studies on the association of AIP with major adverse cardiovascular events did not focus on in the older adults with non-diabetic hypertension, a group which was common in clinical practice but may be underemphasized. This study further demonstrated the prognostic value of AIP in this population. Moreover, SPRINT database was used in this study to further study the relationship between AIP and cardiovascular prognosis under different antihypertensive strategies.

### Study strengths and limitations

This study has the following strengths. First, this study had a sufficient sample size and reliable data source. Second, the population of the study was older adults with non-diabetic hypertension, which was of great need of attention in clinical practice. Finally, the population was grouped according to different antihypertensive strategies to test the consistent of the relationship between AIP and major adverse cardiovascular events.

This study had some limitations. First, the SPRINT trial did not define AIP subgroups, which limited the expansion of the results. Second, some AIP-related data, such as waist circumference and diet, may have altered the study results; this data could not be acquired. Furthermore, an optimal AIP cut-off value for predicting MACEs risks that could aid in finding better risk stratification in clinical settings is yet to be determined.

## Conclusions

In conclusion, this study revealed an independent and positive association between elevated AIP and risk of MACEs among non-diabetic hypertensive older patients. The benefits of intensive BP control in managing cardiovascular events were consistent in the different AIP subgroups. AIP was deemed insignificant for treating hypertensive older patients with intensive BP regimens in clinical settings.

This study found the value of AIP to be a good predictor of cardiovascular prognosis older adults with non-diabetic hypertension. This simple and accessible index can be used to evaluate the prognosis of patients in clinical practice. And in clinical work, AIP can be used as a good prognostic factor, whether patients adopt standard or intensive blood pressure treatment. Future research can pay more attention to the prognostic value of AIP in more different populations or people receiving different treatment strategies.

## Supplementary Information

**Additional file 1: Fig. S1.** Receiver Operator Characteristic Curve for MACES.

## Data Availability

The dataset supporting the conclusions of this article is available in the Biologic Specimen and Data Repository Information Coordinating Center repository, hyperlinked to the dataset at https://biolincc.nhlbi.nih.gov/home/.

## Abbreviations

AIP: Atherogenic index of plasma
MACEs: Major adverse cardiovascular events
BP: Blood pressure
HR: Hazard ratio
TG: Triglyceride
LDL-C: Low-density lipoprotein cholesterol
HDL-C: High-density lipoprotein cholesterol
sdLDL: Small, dense low-density lipoprotein
GGE: Gradient gel electrophoresis ()
SPRINT: Systolic Blood Pressure Intervention Trial
MI: Myocardial infarction
CVD: Cardiovascular disease
CKD: Chronic kidney disease
