# Supplementary material for: Association between the atherogenic index of plasma and major adverse cardiovascular events among non-diabetic hypertensive older adults
Source: Lipids Health Dis. 2022 Jul 22;21:62. doi: 10.1186/s12944-022-01670-6 (PMC9308240; doi:10.1186/s12944-022-01670-6)
Supplement: Supplementary file 1 — Additional file 1: Fig. S1. Receiver Operator Characteristic Curve for MACES. [file 12944_2022_1670_MOESM1_ESM.docx]

Figure S1. Receiver Operator Characteristic Curve for MACES


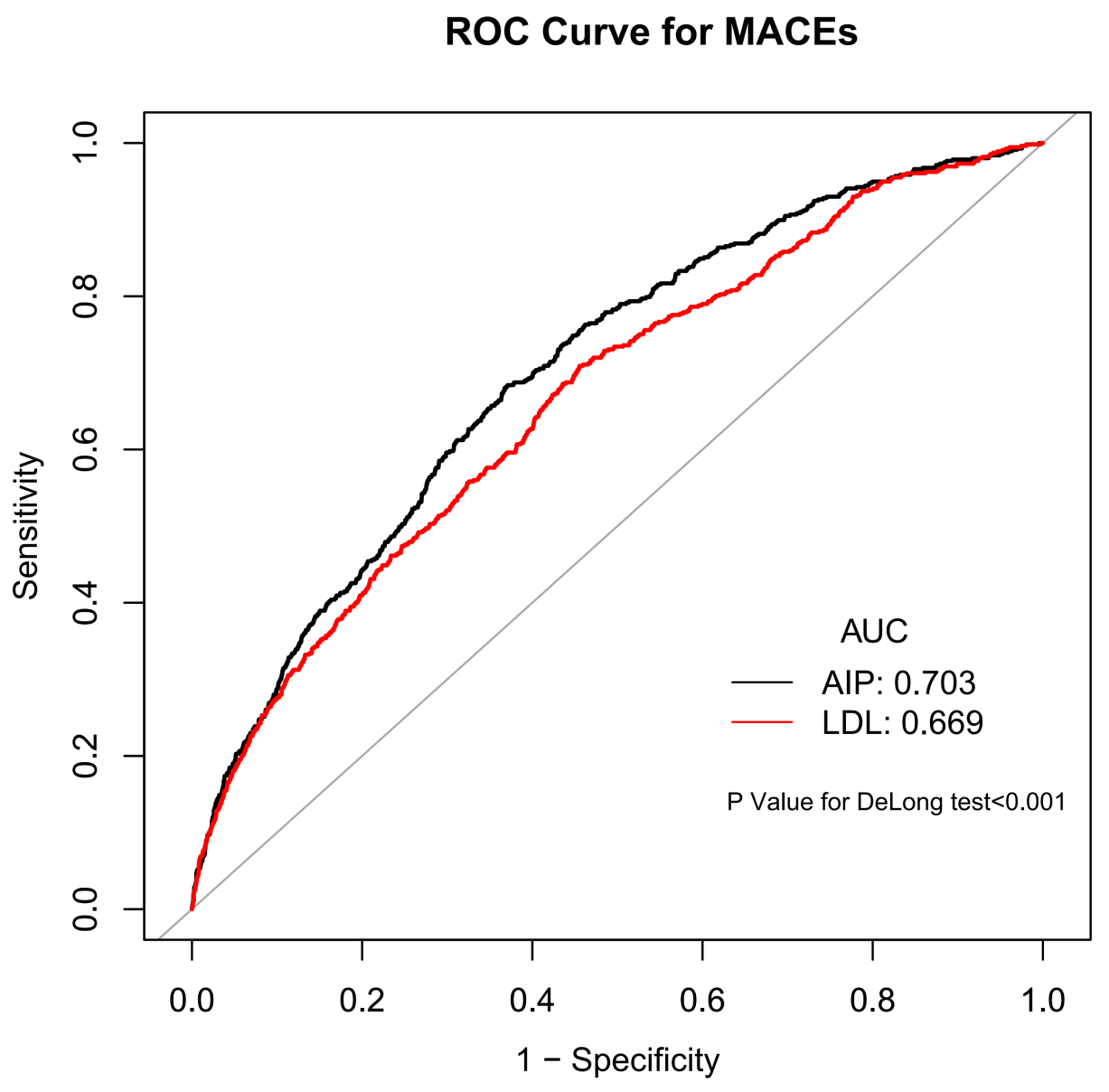


#Other factors in the model included age, race, treatment arms, body mass index, systolic blood pressure, heart rate, smoking status, serum creatinine, fasting total cholesterol, fasting glucose, previous CVD, previous CKD, aspirin use and statin use.
